# (Neo)Adjuvant Treatment of Locally Advanced Esophageal and Gastroesophageal Adenocarcinoma: Special Focus on Sex Differences

**DOI:** 10.3390/cancers14041088

**Published:** 2022-02-21

**Authors:** Thomas Zander, Anna Dorothea Wagner

**Affiliations:** 1 Department I of Internal Medicine, Center for Integrated Oncology Aachen Bonn Cologne Duesseldorf, Gastrointestinal Cancer Group Cologne (GCGC), University of Cologne, 50937 Cologne, Germany; 2 Department of Oncology, Lausanne University Hospital (CHUV), University of Lausanne (UNIL), 1011 Lausanne, Switzerland; dorothea.wagner@chuv.ch

**Keywords:** esophageal cancer, gastroesophageal cancer, sex, locally advanced

## Abstract

**Simple Summary:**

Multimodal therapy is standard in locally advanced esophageal and gastroesophageal adenocarcinoma. Although substantial differences in incidence and outcome between women and men have been observed, no clear sex-specific treatment guide has been developed. In this summary, we described known sex differences focusing on locally advanced esophageal and gastroesophageal adenocarcinoma.

**Abstract:**

Adenocarcinoma of the esophagus and gastroesophageal junction is a common disease. This disease is significantly more prevalent in men, although the main underlying risk factor has an equal sex distribution. In locally advanced disease, multimodal therapy has been developed as the standard in the western world. Neoadjuvant chemoradiotherapy or perioperative chemotherapy using the FLOT regimen was established as the standard. Most recently, adjuvant immunotherapy after neoadjuvant chemoradiotherapy and surgery has been introduced into the multimodal therapy. Substantial sex-specific differences in outcome in multimodal therapy have been described in retrospective subgroup analysis. Further studies are warranted to dissect the sex-specific differences in these treatment regimens.

## 1. Introduction

Esophageal and gastroesophageal junctional (GEJ) carcinoma are among the leading causes of cancer mortality worldwide [1]. In esophageal and GEJ cancer, adenocarcinoma (GEAC) is the predominant histological subtype in the western world. Many patients present at a metastasized stage, but roughly one-quarter of patients with esophageal and GEJ cancer present with localized disease amenable to a curative therapeutic approach. Mortality in the adenocarcinoma population is higher in men (4.9 (4.8–5)/100,000) than in women (0.7 (0.6–0.7)/100,000), and men present more often in an advanced stage (66.5 (65.7–67.2)) than women (59.8 (57.9–61.6)) [1]. The main risk factor of esophageal and GEJ adenocarcinoma is gastric reflux leading to Barrett’s esophagus, the appearance of which is considered a premalignant stage for adenocarcinoma [2]. Whereas reflux is equally present in women and men, erosive reflux disease is more common in men with a men-to-women ratio of 1.6. Even more prominent is the sex difference in progression to Barrett’s metaplasia, with a men-to-women ratio of 1.3 to 4.4, depending on the study population [3]. Several other risk factors were described for esophageal and GEJ adenocarcinoma such as chronic bile reflux, high salt intake, smoking, alcohol intake, and high consumption of nitrates and smoked food [4]. Although sex differences in tobacco and alcohol consumption are well-known, sex differences in the incidence of Barrett’s esophagus or GEJ adenocarcinoma cannot be attributed to these behavioral differences [5]. The other main histological subtype in esophageal cancer is squamous cell cancer, which is more proximally localized and driven by smoking and alcohol consumption. In this summary, we focus on sex differences in neoadjuvant and adjuvant treatment of locally advanced adenocarcinoma of the esophagus and GEJ.

## 2. Results

### 2.1. Neoadjuvant Chemoradiotherapy

The standard for neoadjuvant chemoradiotherapy was defined by the Dutch CROSS trial [6,7]. In total, 368 patients with T2-3, N1 M0, or T1N1 M0 tumors were randomly assigned to chemoradiation (41.4 Gy in addition to weekly carboplatin and paclitaxel) followed by surgery or surgery alone. In the long-term follow-up with a median follow-up time of 147 months, a 13% improvement in overall survival was observed, which included both squamous cell (25%) and adenocarcinoma (75%). Among patients with adenocarcinoma, median overall survival was 43.2 months, and median survival in favor of chemoradiotherapy was 27.1 months (HR 0.73, 95% CI 0.55–0.98, *p* = 0.038). Of note, in this trial, among 366 patients included in the analysis, only 80 were women while 286 (78%) were men [6,8]. While the percentage of men with adenocarcinoma in this trial was not provided, in other Dutch series, over 80% of patients with esophageal adenocarcinoma were men [9]. In the initial publication of the CROSS trial in 2012, the magnitude of benefit from neoadjuvant treatment (adjusted HR) was 0.614 (0.447–0.845, *p* = 0.003) in men compared to 0.928 (0.487–1.766, *p* = 0.82) in women (Table 1). Further efforts were made to improve the neoadjuvant chemoradiotherapy regimen.

In the NeoSCOPE trial, patients were randomized to receive either capecitabine and oxaliplatin with radiotherapy or carboplatin and paclitaxel with radiotherapy. Patients in both arms additionally received two cycles of induction chemotherapy. In this phase II trial, improved overall survival in the carboplatin and paclitaxel arm was observed (HR 0.48, 95% CI 0.24–0.95, *p* = 0.035) [10], underlining the validity of the CROSS regimen. In contrast to the CROSS trial, women demonstrated significantly worse overall survival (Table 1) [10].

Neoadjuvant chemoradiation was further evaluated by a large retrospective analysis, in which 12,460 patients were analyzed of whom 580 received induction chemotherapy in addition to chemoradiotherapy. In this retrospective analysis, induction chemotherapy appeared to improve survival (HR 0.82, 95% CI 0.74–0.92, *p* < 0.001). Furthermore, female sex was associated with an improved outcome. Overall, 12,460 patients were analyzed of whom 12.1% were women. Women had an improved outcome compared to men, with a hazard ratio of 0.87 (0.8–0.92, *p* < 0.001). In concordance with better survival, a significantly higher number of pathologic complete responses was observed [19].

### 2.2. Perioperative Chemotherapy

Perioperative chemotherapy was introduced in the MAGIC trial, in which 503 patients with resectable gastric cancer (74%) or GEJ (14.5%) or esophageal (11.5%) adenocarcinoma were randomized to receive either perioperative chemotherapy (epirubicin–cisplatin–5-FU) followed by surgery or surgery alone. Perioperative chemotherapy improved overall survival (HR 0.75, 95% CI 0.60–0.93, *p* = 0.009). The toxicity of preoperative chemotherapy was manageable, but only 40% of patients were able to complete adjuvant therapy [11]. There was no significant difference in outcome between women and men, although women did benefit more from perioperative treatment (Table 1). Similarly, the FNCLCC/FFCD study using perioperative cisplatin–5-FU chemotherapy demonstrated improved overall survival with multimodal therapy (HR 0.65, 95% CI 0.48–0.89, *p* = 0.003). In contrast to the MAGIC trial, a relatively high proportion of GEJ (64%) and esophageal (11%) adenocarcinomas was recruited in this trial [12]. Further efforts were made to improve the concept of perioperative chemotherapy: in the OE05 trial, chemotherapy was intensified by using four cycles of ECX versus two cycles of cisplatin–5-FU without demonstrating a benefit in overall survival (HR 0.9, 95% CI 0.77–1.05, *p* = 0.19) [20]. In addition to differences in toxicity, a pooled analysis showed that a higher percentage of men (87% vs. 81%, *p* = 0.001) compared to women completed the planned number of neoadjuvant chemotherapy cycles (Table 1) [21]. The observation that women receive overall less chemotherapy is in line with palliative treatment [22]. In addition, women had a significantly higher incidence of serious adverse events during treatment. Nevertheless, despite lower rates of chemotherapy completion, women with localized gastric cancer demonstrated significantly better overall survival (OS) (HR for OS 0.78 (0.70–0.88, *p* < 0.001)) and disease-specific (DSS) survival (HR 0.78 (0.69–0.88, *p* < 0.001)) than men [21]. Furthermore, according to another pooled analysis in patients with metastatic gastric cancer, adjusted OS (HR 0.83, 95% CI 0.72–0.96, *p* = 0.011) in the multivariate model was significantly better in women than in men [22]. In a large metanalysis based on individual patient data from the OE2, MAGIC, OE5, and ST03 trials, sex-specific outcomes were analyzed in more detail. Of 3265 patients included in this analysis, 2627 were men and 597 were women [21]. In this analysis, women showed an improved survival outcome (HR 0.78 (0.70–0.88; *p* < 0.001). This was also reflected by a superior pathological remission rate in women.

Recently, the combination of 5-FU, leucovorin, oxaliplatin, and docetaxel (FLOT) was introduced for the perioperative treatment of patients with gastric and GEJ adenocarcinoma in the AIO-FLOT4 study. In this trial, 716 patients with ≥T2 tumors and/or nodal involvement (N+) were randomly assigned to the chemotherapy regimen established in the MAGIC trial (ECX and ECF) or FLOT. Patients treated with FLOT demonstrated statistically significant and clinically relevant superior overall survival (HR 0.77, 95% CI 0.64–0.94, *p* = 0.012). In addition, increases in R0 resections (85 vs. 78%) and complete pathological response (16 vs. 6%) for patients treated with FLOT were observed. This trial defined FLOT as a new standard for perioperative chemotherapy in the western world. There was no significant difference in outcome between women and men. In the subgroup analysis of women, a small difference in the hazard ratio of 0.8 for women versus 0.76 for men was observed for the FLOT compared to the ECX and ECF regimen (Table 1). The benefit of perioperative chemotherapy was confirmed in an East Asian population in the PRODGY trial: 530 patients with T4, N+ gastric, or GEJ adenocarcinoma were randomized to receive either a combination of neoadjuvant docetaxel, oxaliplatin, and S-1 followed by surgery and adjuvant S-1 or surgery directly followed by S-1. Although overall survival data are not yet mature, a significant improvement in progression-free survival was observed (HR 0.7, 95% CI 0.52–0.95, *p* = 0.023) for patients treated with neoadjuvant chemotherapy (Table 1). This advantage was especially marked in women (HR 0.5, 95% CI 0.27–0.91). No strong safety signal was observed with grade ≥ 3 toxicity of 6.3% in either arm [14]. Similarly, in the Chinese RESOLVE trial, the concept of perioperative chemotherapy was evaluated in gastric and GEJ cancer. A total of 1022 patients with cT4 tumors were randomized to receive either adjuvant XELOX, adjuvant SOX, or neoadjuvant SOX followed by adjuvant S-1. The study met its primary endpoint of demonstrating the superiority of neoadjuvant chemotherapy with a three-year disease-free survival of 62% for the neoadjuvant arm compared to 54.8% for the standard arm (HR 0.79, 95% CI 0.62–0.99, *p* = 0.045). Non-inferiority of adjuvant XELOX compared to adjuvant SOX was additionally demonstrated in this trial [23,24].

### 2.3. Adjuvant Chemoradiotherapy

Adjuvant chemoradiotherapy has mainly been evaluated in gastric cancer in several trials, some of which also included GEJ patients. In the INT-0116 trial, 556 patients with gastric cancer were randomly assigned to receive either surgery alone or surgery followed by chemoradiotherapy (45 Gy and 5-FU). In this trial, overall survival was improved by adjuvant chemoradiotherapy (HR 1.35, 0.5% CI 1.09–1.66, *p* = 0.005) [15]. Ninety percent of the patients in the trial had a D0/1 lymph node resection. For this reason, the benefit of adjuvant chemoradiation after D2 resection remained unclear after this trial. When analyzed in more detail, women seemed not to benefit from adjuvant treatment. Women with diffuse-type gastric cancer treatment even showed inferior survival when treated with adjuvant chemoradiotherapy (Table 1) [25].

Starting from this observation, in the CRITICS trial, adjuvant chemotherapy was compared to adjuvant chemoradiation after neoadjuvant chemotherapy and surgery. No difference in overall survival was observed (HR 1.01, 95% CI 0.84–1.22, *p* = 0.9). Again, a large number of patients in this trial was not able to complete adjuvant chemotherapy (50%), underlining the toxicity of this therapy after neoadjuvant chemotherapy and surgery [16]. In this trial, an important sex disparity in survival between the treatment arms was observed: while men showed better survival when treated with chemoradiotherapy (HR 1.30, 0.55 CI 0.96–1.76), women showed better survival when treated with adjuvant chemotherapy (HR 0.68, 95% CI 0.44–1.04) (Table 1). Again, the reasons for this observation are currently unclear. It confirms the need for further research on this topic [16].

### 2.4. New Drugs

#### 2.4.1. Angiogenesis Inhibitors

Comprehensive sequencing and functional analysis have led to an improved biological understanding of esophageal and GEJ adenocarcinoma. In the past few years, several new drugs have been first evaluated in the palliative setting and later studied in the locally advanced stage. In the ST03 trial, 1063 patients with gastric and GEJ cancer were included and treated with perioperative chemotherapy (ECX and ECF), with or without additional bevacizumab. No benefit was observed in overall survival, neither in the overall population nor any particular subgroup (HR 1.08, 95% CI 0.91–1.29 *p* = 0.36), with more perioperative grade ≥ 3 toxicity in the experimental arm [17]. No survival difference was observed between men and women. Similarly, evaluating the addition or ramucirumab to FLOT in the FLOT7-RAMSES trial, slightly more anastomotic leakage was observed in patients treated with ramucirumab [26], and sex-specific outcomes were not reported. Bevacizumab was also combined with chemoradiotherapy in phase II trials, but no clear improved outcome was observed [27,28] and the numbers were too small to detect sex differences.

#### 2.4.2. ERBB2-Targeting Drugs

Overexpression of HER2 or amplification of ERBB2 was described in gastric and GEJ tumors in about 10% of the patients [29]. Overexpression is seen more often in men than in women (OR 1.48, 95% CI 1.34–1.65) [30,31]. Trastuzumab was introduced for the treatment of metastasized gastric and GEJ tumors more than a decade ago [32]. In this trial, no significant difference between men and women in the benefit from the trastuzumab addition was reported.

For patients with HER2-positive gastric cancer, initial trials demonstrated the feasibility of adding trastuzumab to FLOT for perioperative therapy of gastric and GEJ cancer. In a single-arm trial, 56 HER2-positive patients were treated with perioperative FLOT and trastuzumab followed by nine cycles of trastuzumab monotherapy. This trial included a high proportion of GEJ tumors (71%). Median disease-free survival of 42.5 months was promising, as well as the three-year overall survival rate of 82.1% [33]. More recently, further agents targeting ERBB2 were evaluated in the locally advanced stage. In the TRAP phase II trial, 40 patients were assigned to receive standard chemoradiotherapy according to the CROSS trial and additional trastuzumab and pertuzumab. In this trial, no unexpected safety signals were observed. In a propensity-matched analysis, survival appeared superior compared to historical data (HR 0.58, 95% CI 0.34–0.97) [34]. The targeting of ERBB2 was also evaluated in combination with perioperative chemotherapy. In the PETRARCA trial, patients received trastuzumab and pertuzumab in addition to perioperative FLOT. A total of 81 patients were randomized in this phase II trial. Significantly higher pathological remission rates (35 vs. 12%, *p* = 0.02) and a higher rate of lymph node negativity (68 vs. 39%) were seen in patients receiving additional trastuzumab and pertuzumab. Follow-up was still too short to evaluate DFS and OS [33,35]. Other trials addressing this question are ongoing [36]. At present, patient numbers in these trials are too small to draw any conclusions regarding sex differences in outcomes.

#### 2.4.3. PDL1/PD1 Inhibitors

Immunotherapy interrupting the PD1–PDL1 interaction demonstrated clinical benefit in metastasized adenocarcinoma of the GEJ with slightly less benefit for women [37]. Further efforts were made to implement immunotherapy in the treatment of locally advanced esophageal and GEJ adenocarcinoma. Atezolizumab was added to standard chemoradiotherapy according to the CROSS trial in the PERFECT trial. In total, 40 patients were enrolled. Chemo-immuno-radiotherapy was demonstrated to be feasible, and an INFy signature was identified to predict clinical response [38]. Feasibility was further underlined by a retrospective analysis of 168 patients of whom 25 received chemo-immuno-radiotherapy in neoadjuvant intention. No difference in perioperative mortality was observed in patients with additional immunotherapy [39,40]. Again, patient numbers in these analyses were too small to allow any conclusions regarding potential sex differences in outcomes. Most recently, adjuvant immunotherapy was evaluated in the CheckMate 577 trial [18]. In this global trial, 794 patients with esophageal or GEJ carcinoma (both adeno- and squamous cell) with residual tumor after neoadjuvant chemoradiotherapy and surgery were randomized 2:1 to adjuvant nivolumab for 1 year or placebo. The primary endpoint of the trial was disease-free survival. Overall, with a median disease-free survival of 22.4 vs. 11.0 months (HR 0.69, 95% CI 0.56–0,86), this trial met its primary endpoint. Additionally, in the subpopulation of adenocarcinoma, significantly greater disease-free survival for patients treated with adjuvant nivolumab was observed (HR 0.75, 95% CI 0.59–0.96). One major risk after neoadjuvant chemoradiotherapy is distant metastases. Adjuvant immunotherapy increased the time to distant metastases (HR 0.74, 95% CI 0.6–0.92). At the same time, no deterioration in patient-reported outcome was observed. Women appeared to have an even larger benefit from adjuvant nivolumab (HR 0.59, 95% CI 0.35–1.00) compared to men (HR 0.73, 95% CI 0.59–0.91) (Table 1) [18]. A more detailed subgroup analysis is still missing. In a systematic review of PD1–PDL1 inhibition in upper GI cancer, women tended to show less benefit (HR 0.99, 95% CI 0.8–1.22) than men (0.82, 95% CI 0.69–0.97) [41].

## 3. Discussion

Sex-specific differences in outcomes have been observed in several clinical trials but have not been reported in all trials. Many of the trials discussed were too small to draw any conclusions regarding sex-specific outcomes. In several of the trials on neoadjuvant chemoradiation, clinically relevant differences in outcomes were observed between women and men. For the CROSS trial, e.g., the benefit from neoadjuvant treatment in patients with adenocarcinoma was lower than in patients with squamous cell cancer. This trial was neither designed nor powered to detect treatment effects in men and in women separately, and no definitive conclusions can be drawn from the observation that women did not benefit from neoadjuvant chemoradiation. It illustrates the need to consider not only potential differences in treatment outcomes between patients with adeno- and squamous cell carcinomas but also potential differences in treatment effects between men and women in future clinical trials, as they may reflect differences in tumor biology between cancers of the same (sub)type. Furthermore, it illustrates the necessity to conduct exploratory analyses controlling for sex not only for the primary outcome but also for subgroups and secondary outcomes.

In general, for systemic treatment, sex differences in outcomes can be attributed to either differences in pharmacokinetics and/or pharmacodynamics [42]. 5-fluouracil (5-FU) is the backbone of the majority of chemotherapy regimens for gastrointestinal cancers. It is a classic example of a drug that shows about a 20% higher clearance in men [43] independent of age [44] and results in significantly higher plasma levels in women [43,44]. Furthermore, the clearance of SN-38, the active metabolite of irinotecan, is 30–38% lower in women [45]. In addition, the large interpatient variability in the clearance of 5-FU is not reduced by the current practice to adapt chemotherapy according to the body surface area [46]. Apart from differences in pharmacokinetics, differences in tumor biology between women and men, also referred to as sexual dimorphism in cancer [47,48], for non-sex-related cancers might explain differences in outcomes for both systemic treatments and radiotherapy.

In addition to differences in the efficacy and toxicity of systemic treatments, sex differences in the frequency of perioperative complications were observed in large cohort studies: in a recently published national cohort study including 4937 surgically treated patients with gastroesophageal adenocarcinomas from the Netherlands [9], men demonstrated significantly higher rates of cardiac comorbidities than women. The different clustering of comorbidities between men and women is likely to contribute to the different rates of surgical complications, with a significantly greater risk of complications in men (38.1 vs. 32.9%, *p* = 0.017). In addition, men had more pulmonary complications (15.7 vs. 10.8%, *p* = 0.002), anastomotic leakages (7.5 vs. 5.1%, *p* = 0.031), and re-interventions (16.2 vs. 11.9, *p* = 0.008) after surgery for gastroesophageal adenocarcinomas.

Sex differences in response to PDL1–PD1 inhibition have been described in several cancer entities with women generally having a smaller benefit [49]. Further research is necessary to confirm or infirm this observation and, if confirmed, understand the reasons behind this observation.

In summary, sex is an important clinical variable that can significantly impact the outcome of patients with locally advanced cancers of the esophagus and GEJ. However, comprehensive evaluations of its impact on all types of treatments of these diseases are missing and urgently needed.

## 4. Conclusions

Locally advanced adenocarcinoma of the esophagus and the gastroesophageal junction warrants interdisciplinary and multimodal discussion and treatment. Sex-specific differences in outcome have been observed in a number of trials, but substantial research must be conducted to understand their biological basis and tailor therapies accordingly. A large number of trials investigating targeted therapies and immunotherapy are ongoing.

## Figures and Tables

**Table 1:** Randomized trials on multimodal therapy in esophageal and gastroesophageal cancer.

Trial (REF)	Pts (f:m)	OS (HR; 95% CI; *p*-Value)	PFS/DFS (HR)	Women OS	Men OS	Women PFS	Men PFS
CROSS [6]	366 (80:286)	0.665; (0.500–0.884); *p* = 0.003	0.498; (0.357–0.693); *p* < 0.001	0.928; (0.487–1.766); *p* = 0,82	0.614; (0.447–0.845); *p* = 0.003	Not reported	Not reported
NeoSCOPE [10]	85 (16:69)	0.48; (0.24–0.95); *p* = 0.035	0.54; (0.29–1.01); *p* = 0.053	Not reported in detail but not significant	Not reported in detail but not significant	Not reported but not significant	Not reported but not significant
MAGIC [11]	503 (107:396)	0.75; (0.6–0.93); *p* = 0.009	0,66; (0,53–0,81); *p* < 0.001	Death/treated 23/45 vs. 3/62	Death/treated 126/205 vs. 127/191	Not reported	Not reported
FNCLCC/FFCD [12]	224 (37:187)	0.69; (0.50–0.95); *p* = 0.02	0.65; (0.48–0.89); *p* = 0.003	Not reported	Not reported	Not reported	Not reported
FLOT [13]	716 (183:533)	0.77; (0.63–0.94); *p* = 0.012	0.75; (0.62–0.91); *p* = 0.0036	0.8 (ns)	0.76 (ns)	Not reported	Not reported
PRODGY [14]	484 (100: 384)	0.70; (0.52–0.95); *p* = 0.023	0.84; (0.60–1.19); *p* = 5.338	0.73 (0.37–1.45) ns	0.83 (0.55–1.26) ns	0.5; (0.27–0.91); significant	0.77; (0.54–1.12); not significant
INT-0116 [15]	556 (30:70%)	1.35; (1.09–1.66); *p* = 0.005	1.52; (1.23–1.86); *p* < 0.001	Not reported	Not reported	Not reported	Not reported
CRITICS [16]	788 (259:529)	1.01; (0.84–1.22); *p* = 0.90	0.99; (0.82–1.19); *p* = 0.92	0.68; (0.44–1.04)	1.30; (0.96–1.76)	Not reported	Not reported
ST03 [17]	1063 (204:859)	1.09 (0.91–1.29)	1.05; (0.89–1.23); *p* = 0·56	1.07; (0.70–1.65)	1.08; (0.89–1.31)	Not reported	Not reported
577 [18]	794 (123:671)		0.69; (0.56–0.86); *p* < 0.001	Not reported	Not reported	0.59; (0.35–1.00)	0.73; (0.59–0.91)
